# Effect of 3-HBI on Liver Fibrosis via the TGF-β/SMAD2/3 Pathway on the Human Hepatic Stellate Cell Model

**DOI:** 10.3390/ijms26136022

**Published:** 2025-06-23

**Authors:** Chavisa Khongpiroon, Watunyoo Buakaew, Paul J. Brindley, Saranyapin Potikanond, Krai Daowtak, Yordhathai Thongsri, Pachuenp Potup, Kanchana Usuwanthim

**Affiliations:** 1 Cellular and Molecular Immunology Research Unit, Faculty of Allied Health Sciences, Naresuan University, Phitsanulok 65000, Thailandkraid@nu.ac.th (K.D.); yordhathait@nu.ac.th (Y.T.); pachuenp@nu.ac.th (P.P.); 2 Department of Microbiology, Faculty of Medicine, Srinakharinwirot University, Bangkok 10110, Thailand; watunyoo@g.swu.ac.th; 3 Department of Microbiology, Immunology and Tropical Medicine, School of Medicine & Health Sciences, George Washington University, Washington, DC 20037, USA; pbrindley@email.gwu.edu; 4 Department of Pharmacology, Faculty of Medicine, Chiang Mai University, Chiang Mai 50200, Thailand; saranyapin.p@cmu.ac.th

**Keywords:** 3-HBI, liver fibrosis, TGF-β/SMAD2/3 pathway

## Abstract

Liver fibrosis can progress to irreversible cirrhosis if the underlying causes remain, and this can in turn develop into hepatocellular carcinoma (HCC). Despite these adverse outcomes, liver fibrosis can be reversed. Consequently, research has focused on substances that target liver fibrosis to prevent or reduce its progression. This study deals with the potential anti-fibrotic action of 3-hydroxy-β-ionone (3-HBI), a bioactive compound found in many plants. To assess the putative effects of 3-HBI, pro-inflammatory cytokine production and the expression of genes and proteins associated with the TGF-β/SMAD2/3 pathway were monitored following exposure to 3-HBI. Initially, cells of the human hepatic stellate cell line LX-2 were treated with TGF-β1 to simulate fibrogenesis. Following the exposure of activated LX-2 cells to 3-HBI, the production of pro-fibrotic substances was significantly reduced. Molecular docking studies revealed that 3-HBI exhibited a high binding affinity for key proteins in the TGF-β/SMAD2/3 pathway. Analyses using qRT-PCR and Western blotting revealed that 3-HBI suppressed the expression of TIMP1, MMP2, MMP9, COL1A1, COL4A1, SMAD2, SMAD3, SMAD4, MMP2, and ACTA2. Together, these findings demonstrate that 3-HBI inhibited the activation of LX-2 cells and significantly reduced the proinflammatory responses triggered by TGF-β1. Accordingly, we confirmed the noteworthy potential of 3-HBI as a therapeutic agent to prevent and treat liver fibrosis, effected by its modulation of the TGF-β/SMAD2/3 signaling pathway.

## 1. Introduction

Liver fibrosis, which is considered a precursor to irreversible cirrhosis, results from persistent liver injury, and predisposes individuals to severe complications such as portal hypertension, hepatic encephalopathy, and hepatocellular carcinoma (HCC). Characterized by excessive scar tissue formation, liver fibrosis disrupts the normal liver architecture and function, progressively impairing its hepatic performance [1]. Despite the severity of these complications, liver fibrosis retains the potential for reversibility, particularly if detected early and depending on the underlying causative factors. Thus, research efforts have mainly been toward the investigation of drugs that target liver fibrosis and potentially impede or block the advancement of liver fibrosis.

Hepatic fibrosis is a wound-healing process caused by chronic liver injury. In the pathophysiology of liver fibrosis, an imbalance in the synthesis and breakdown of the extracellular matrix (ECM) leads to the accumulation of the ECM, including type I collagen (COL1A1) and hyaluronic acid. Hepatic fibrosis brought on by inflammatory reactions that damage liver tissue is a typical occurrence in the majority of chronic liver disorders. Prolonged inflammation eventually leads to the development of scar tissue, which is referred to as fibrosis. Hepatic stellate cells (HSCs) are the primary effector cells in liver fibrosis. They are activated by damage and transdifferentiate into myofibroblast-like cells, which secrete excessive ECM components, contributing to the accumulation of scar tissue [2,3]. Once activated, HSCs release inflammatory agents that cause liver fibrosis. The upregulation of fibrogenic cytokines, including transforming growth factor beta (TGF-β), is closely associated with the development of liver fibrosis. TGF-β is a key signaling molecule that plays a critical role in liver fibrosis by promoting fibrogenesis. It stimulates the activation and proliferation of HSCs, which are normally quiescent cells in the liver. Furthermore, TGF-β inhibits ECM degradation, thereby enhancing fibrosis progression [4,5]. Novel drugs are needed, since specific treatments for hepatic fibrosis are not currently available. The treatment options for liver fibrosis include blocking the TGF-β/mothers against the decapentaplegic homolog 2 and 3 (SMAD 2/3) signaling pathway and focusing on HSC activation. By blocking TGF-β signaling, it is possible to reduce HSC activation, limit ECM accumulation, and attenuate the inflammatory environment, ultimately slowing or preventing the progression of liver fibrosis.

**Figure 1:**
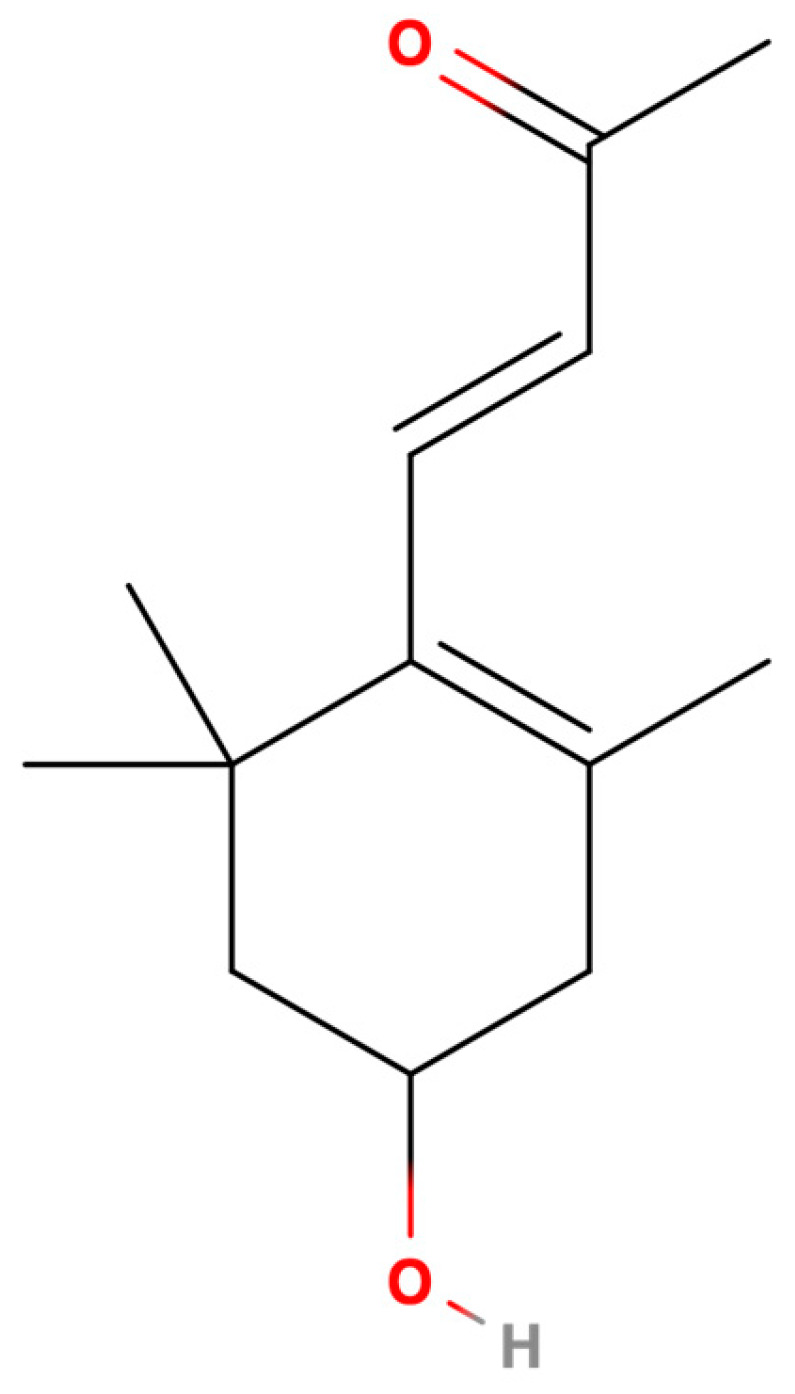
Structure of 3-hydroxy-β-ionone (3-HBI).

3-Hydroxy-β-ionone (3-HBI) has been identified in *Moringa oleifera* Lam. [6] as well as the rice cultivar Kartikshail [7] and the pleurocarpous moss *Rhynchostegium pallidifolium* [8] from Bangladesh. Figure 1 shows the structure of 3-HBI. It has been shown to have anti-inflammatory [6,9] and anti-cancer [10,11,12] properties. However, information on the fundamental mechanism of 3-HBI on hepatic fibrosis has yet to be reported. Our purpose here was to investigate the potential of 3-HBI for the treatment of hepatic fibrosis. Using a model of liver fibrosis and focusing on the TGF-β/SMAD2/3 signaling pathway, we observed that the exposure of TGF-β1-activated HSC to 3-HBI downregulated the pro-inflammatory cytokines ACTA2, TIMP1, COL1A1, COL4A1, SMAD2, SMAD3, SMAD4, MMP2, and MMP9. These findings highlight the potential that 3-HBI exhibits as a therapeutic substance for liver fibrosis.

## 2. Results

### 2.1. The Effect of 3-HBI on LX-2 Cell Viability

**Figure 2:**
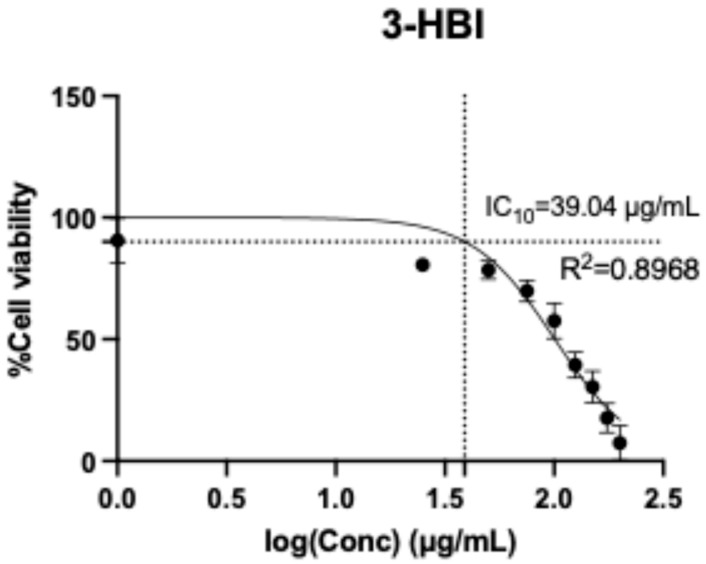
The cytotoxicity of 3-HBI on LX-2 cells according to a resazurin reduction assay in the concentration range of 0–200 μg/mL.

HSC activation is integral to hepatic fibrosis. This study used LX-2 cells, which are classified as an HSC cell line. 3-HBI was dissolved in a mixture of Tween 80 and DMSO. A resazurin reduction assay was used to measure the cytotoxicity of 3-HBI on LX-2 cells at concentrations ranging from 0 to 200 μg/mL. After 24 h of incubation, the 10 percent inhibitory concentration (IC10) of 3-HBI on LX-2 was 39.04 μg/mL (Figure 2). Concentrations of 5 and 10 μg/mL of 3-HBI were selected for further investigation.

### 2.2. The Effects of 3-HBI on Expression of Liver Fibrotic Markers

**Figure 3:**
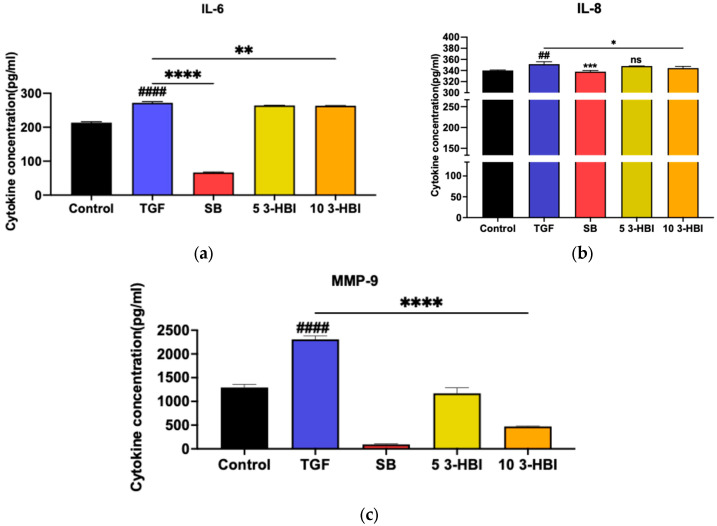
The cytokine production of (**a**) IL-6, (**b**) IL-8, and (**c**) MMP-9 by LX-2 cells. Control: untreated; TGF-β: transforming growth factor beta, 10 ng/mL; SB: SB431542, 10 mM; 5 3-HBI: 3-HBI, 5 μg/mL; 10 3-HBI: 3-HBI, 10 μg/mL. Data are represented as means ± SD. ^##^ *p* < 0.01 and ^####^ *p* < 0.01 compared to control; * *p* < 0.05, ** *p*< 0.01, *** *p* < 0.001, and **** *p* < 0.0001 compared to TGF-β1-treated group; ns: not significant.

This study examined whether 3-HBI could reduce liver fibrosis in LX-2 cells by detecting its impact on the production of cytokines and the extracellular matrix (ECM) (IL-6, IL-8, and MMP-9). LX-2 cells were induced with 10 ng/mL of TGF-β and treated with 3-HBI for 24 h. The supernatant was collected, and the levels of cytokines and the ECM were measured by ELISA. The secretion of IL-6, IL-8, and MMP-9 was higher in LX-2 cells stimulated by TGF-β than in LX-2 cells not stimulated by TGF-β. In contrast, when treated with SB431542 (a specific small-molecule inhibitor that targets TGF-β), LX-2 cells exposed to 3-HBI secreted less IL-6, IL-8, and MMP-9 than those not treated with SB431542 (Figure 3a–c).

### 2.3. 3-HBI Suppresses Fibrotic Markers and Inhibits HSC Activation by Blocking SMAD Signaling Pathway at the Gene Expression Level

**Figure 4:**
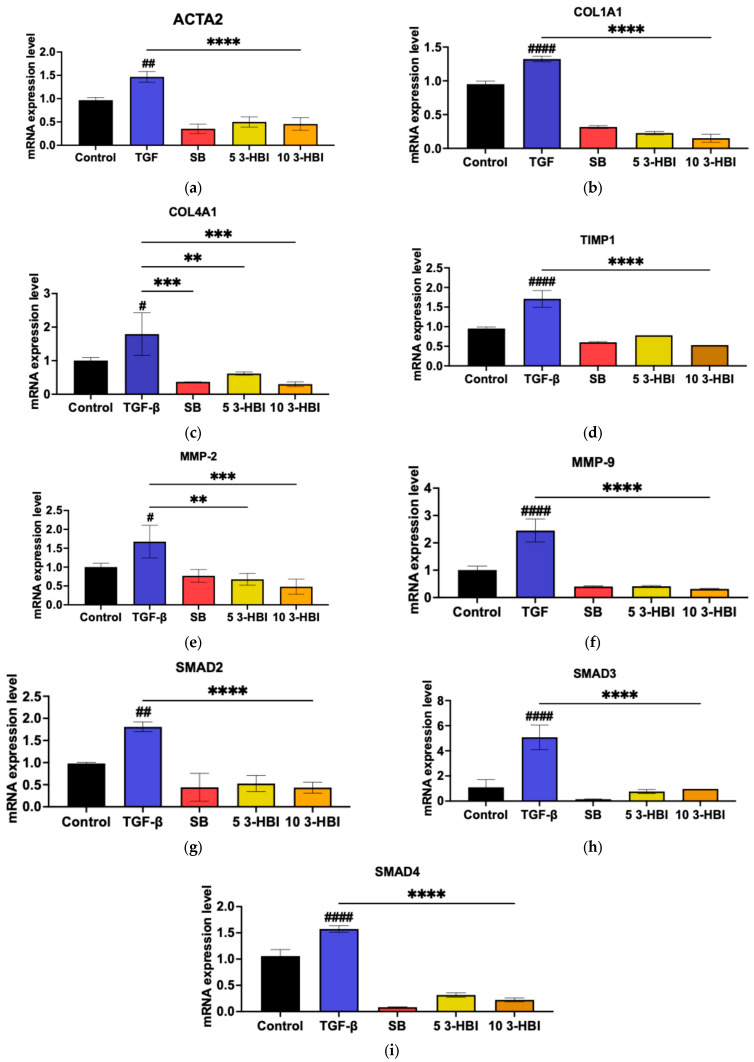
Effects of 3-HBI on mRNA expression. The expression of (**a**) ACTA2, (**b**) COL1A1, (**c**) COL4A1, (**d**) TIMP1, (**e**) MMP2, (**f**) MMP9, (**g**) SMAD2, (**h**) SMAD3, and (**i**) SMAD4 was measured by real-time qRT-PCR. GAPDH served as an internal control. Control: untreated; TGF-β: transforming growth factor beta, 10 ng/mL; SB: SB431542, 10 mM; 5 3-HBI: 3-HBI, 5 μg/mL; 10 3-HBI: 3-HBI, 10 μg/mL. Data are represented as means ± SD. ^#^ *p* < 0.05, ^##^ *p* < 0.05 and ^####^ *p* < 0.01 compared to control; ** *p* < 0.01, *** *p* < 0.001, and **** *p* < 0.0001 compared to TGF-β1-treated group.

We postulated that 3-HBI might also inhibit hallmark fibrotic indicators at the gene expression level, considering the finding that it inhibits HSC activation through TGF-β1. The ACTA2, COL1A, COL4A1, TIMP-1, MMP-2, and MMP-9 mRNA expression levels were assessed using real-time qRT-PCR with GAPDH as an internal control to test the hypothesis. Figure 4a–f show that, in comparison to untreated cells, the expression of all the selected genes significantly increased after 48 h of incubation with 10 ng/mL of TGF-β1. Remarkably, 3-HBI inhibited the expression of every gene examined. These findings imply that 3-HBI first inhibits the transcriptional level of fibrotic markers. Consequently, we assessed whether 3-HBI inhibits SMAD2/3, which is triggered by TGF-β1, in order to affect its antifibrotic activity. As observed in Figure 4g–i, the mRNA expression in LX-2 cells of SMAD2, SMAD3, and SMAD4 increased in comparison to the untreated control cells.

### 2.4. 3-HBI Inhibits the Fibrotic Marker Protein via the SMAD2/3 Pathway

**Figure 5:**
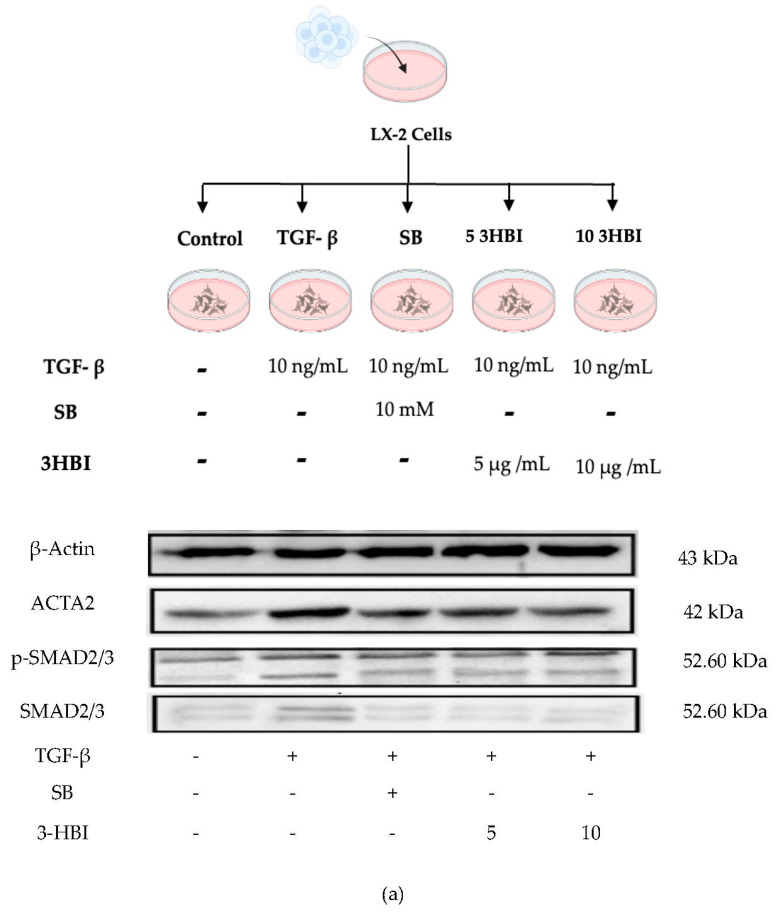

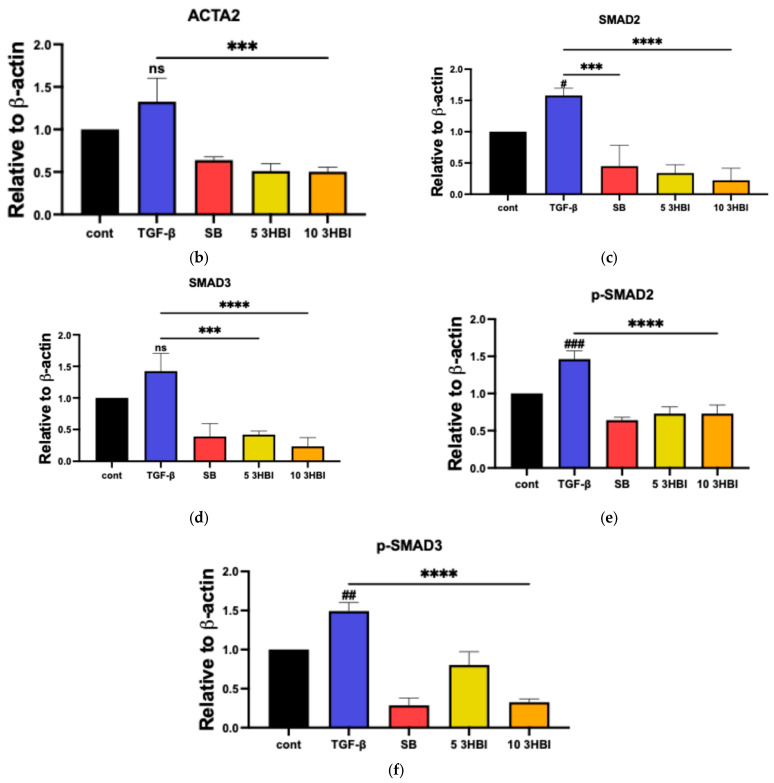
Effects of 3HBI on ACTA2, p-SMAD2/3, and SMAD2/3 protein production. The quantitative protein production of ACTA2, p-SMAD2/3, and SMAD2/3 was measured by Western blotting (**a**). The bar graphs represent the relative expression of these proteins after normalization to β-actin (**b**–**f**). Control: untreated; TGF-β: transforming growth factor beta, 10 ng/mL; SB: SB431542, 10 mM; 5 3-HBI: 3-HBI, 5 μg/mL; 10 3-HBI: 3-HBI, 10 μg/mL. Data are represented as means ± SD. ^#^ *p* < 0.05, ^##^ *p* < 0.01, and ^###^ *p* < 0.01 compared to control; *** *p* < 0.001 and **** *p* < 0.0001 compared to TGF-β1-treated group; ns: not significant.

We verified these results through an immunoblot analysis of the protein levels following 3-HBI treatment, which was related to a decrease in the expression of characteristic fibrosis-related and SMAD-signaling genes, including ACTA2, COL1A1, COL4A1, TIMP-1, MMP-2, MMP-9, SMAD2, SMAD3, and SMAD4. The protein levels of ACTA2, p-SMAD2/3, and SMAD2/3 were low in untreated cells. As seen in Figure 5a, however, the TGF-β1 treatment significantly increased the synthesis of these proteins. Figure 5b–f show that 3-HBI, accordingly, markedly inhibited the TGF-β1-induced synthesis of ACTA2, p-SMAD2/3, and SMAD2/3 in a concentration-dependent manner.

### 2.5. In Silico Molecular Docking Analysis of Candidate Target Proteins

**Figure 6:**
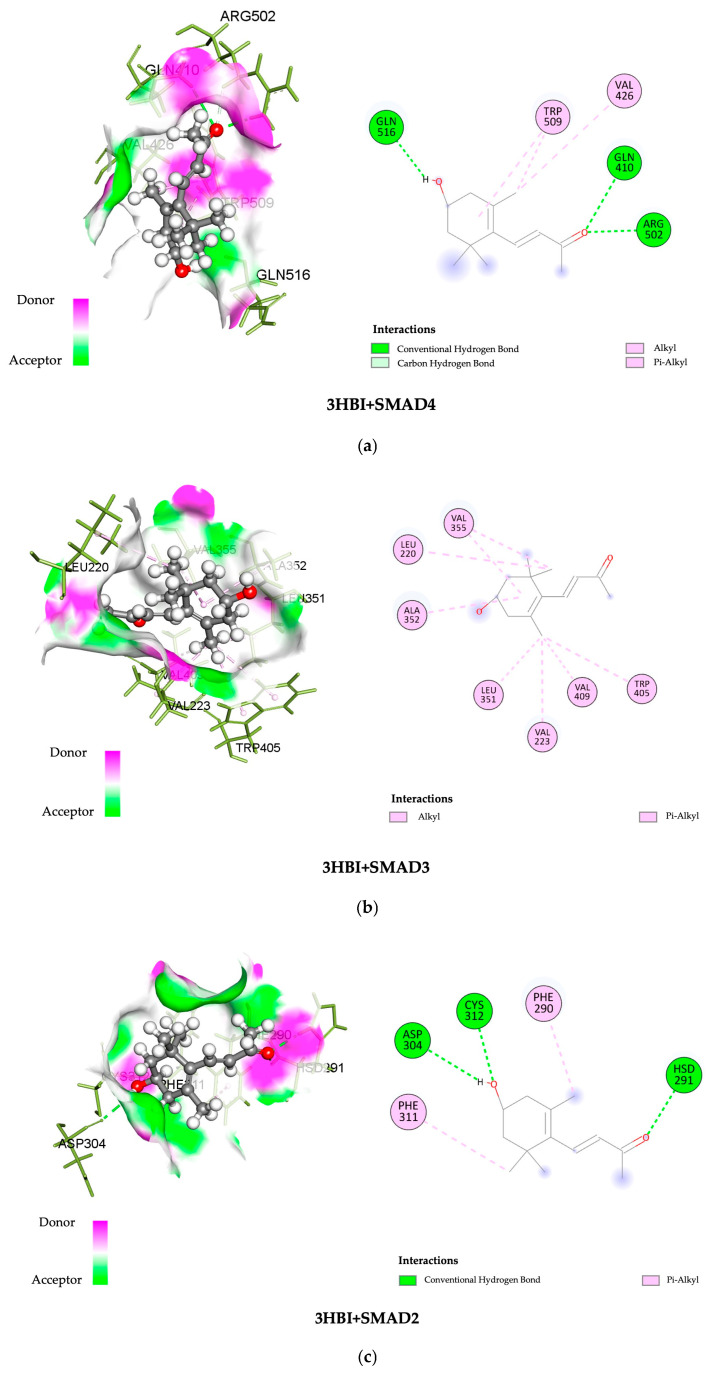

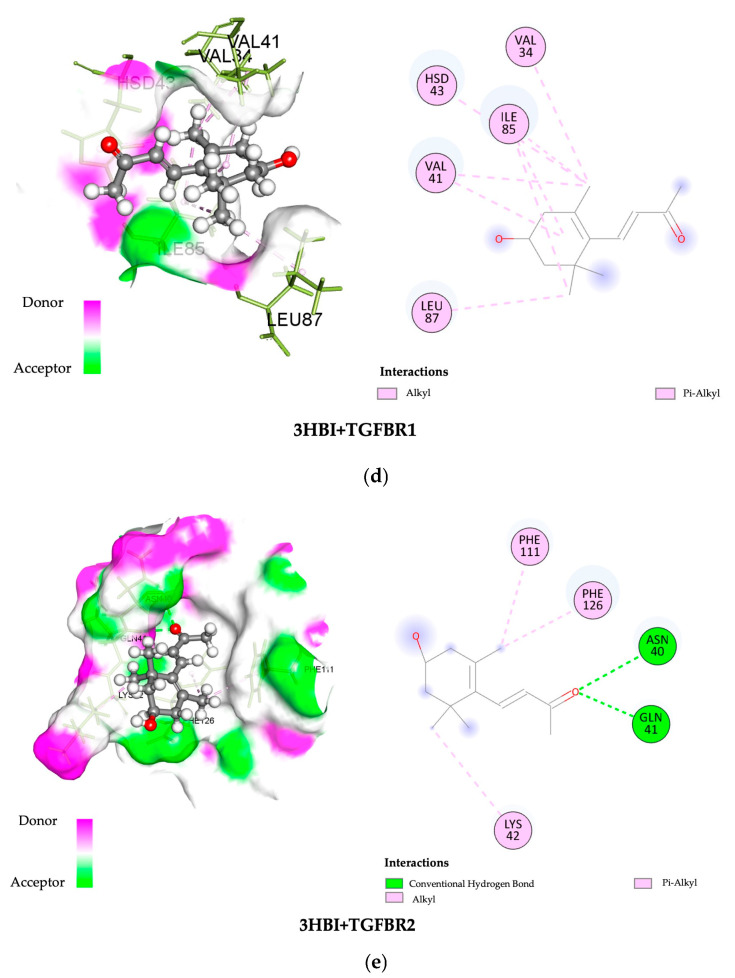
Three-dimensional and two-dimensional images showing the binding of 3-HBI to protein in the SMAD signaling pathway. (**a**) SMAD4, (**b**) SMAD3, (**c**) SMAD2, (**d**) TGF-βR1, and (**e**) TGF-βR2.

The SWISS-dock web-based platform’s cavity-detection-guided blind docking features were used to investigate the binding interactions between 3-HBI and putative protein targets. Table 1 provides specific findings, such as the Autodock Vina binding scores and the amino acids involved in the interactions. In Figure 6, the protein–ligand interactions are shown in both 2D and 3D formats. Notably, 3-HBI exhibited good binding scores (−3 to −5 kcal/mol), with target proteins known to be involved in the signaling cascade of SMADs. Molecular docking revealed information about the precise interactions between 3-HBI and proteins in the SMAD2/3 pathway, as there are several proteins that bind in the SMAD signaling pathway. This insight was able to determine the chemical processes that underlie cellular signaling, as detailed in Table 1. Figure 6a–e display the structural data (2D and 3D) of the 3-HBI protein docking combination.

## 3. Discussion

The FDA approved resmetirom (Rezdiffra) for the treatment of moderate to advanced fibrosis from noncirrhotic non-alcoholic steatohepatitis (NASH), marking a significant advancement in targeted therapy [13]. Recent scientific advancements offer exciting opportunities. To treat liver fibrosis, new medications must be researched because there are few medications specifically for this condition. Therefore, the development of novel anti-fibrotic medications to expand the limited available options is a medical therapeutic priority. As such, a key objective of the current research in this area is to examine substances that target liver fibrosis and may prevent or slow its progression. In hepatic fibrosis, hepatic stellate cells (HSCs) are key mediators of extracellular matrix (ECM) synthesis. TGF-/SMAD, PDGF, NF-κB, and Wnt/β-catenin are among the signaling cascades and growth factors that coordinate this phenotypic transition in response to pathogenic stimuli associated with liver fibrosis [14,15,16,17,18]. The TGF-/SMAD pathway is the mechanism of interest in liver fibrosis. The pathway follows TGF-1 binding to its related receptor (TGF-receptor 1), and the network and triggering of downstream effectors.

The current study, which examined how 3-hydroxy-β-ionone (3-HBI) affects HSC liver fibrosis in vitro, found that these fibrosis-associated genes were upregulated in HSCs following the activation of the TGF-β/SMAD signaling pathway. Inflammatory markers such IL-6 and IL-8 as well as matrix metalloproteinases were released at higher levels. Conversely, the 3-HBI treatment for fibrosis led to the decreased expression of mRNA and protein markers related to fibrosis (Figure 3, Figure 4 and Figure 5). This suggests that 3-HBI interferes with HSC activation through the disruption of the TGF-β/SMAD signaling cascade, through downregulating the production of SMAD2 and SMAD3. The findings for 3-HB1 coincide with those of earlier reports on other natural compounds, including β-citronellol from *Citrus histrix* DC. [17], 1-phenyl-2-pentanol and oleamide from *Moringa oleifera* Lam. [16,19], and stem extract from *Salacia chinensis* L. [20], to target these molecular pathways and block the development of liver fibrosis. The use of these three herbs in folk medicine to treat inflammation and liver illness corresponds to the demonstration of the ability of natural products to inhibit fibrotic markers, such as the production of pro-inflammatory cytokines, and reduce the synthesis of the extracellular matrix. In the current study, it was confirmed that 3-HBI, a bioactive chemical present in *Moringa oleifera* Lam. leaves, can downregulate the pro-inflammatory cytokines MMP2, MMP9, ACTA2, TIMP1, COL1A1, COL4A1, SMAD2, SMAD3, and SMAD4. These results demonstrate 3-HBI’s potential as a liver fibrosis treatment.

Additionally, a previous study that explored other pathways leading liver fibrosis found that pantoprazole was significantly fibrogenic, both in vitro and in vivo, by inhibiting the deubiquitinating enzyme OTUB2 and the Yes-associated protein signaling pathway [21]. Ruxolitinib, a small molecule that directly targets Janus kinase 1/2 and slows the progression of liver fibrosis, has, however, demonstrated promising anti-fibrotic benefits in both in vitro and in vivo tests [22]. Vatalanib, a tyrosine kinase inhibitor that acts on VEGF receptors, inhibits angiogenesis, which in turn reduces liver fibrosis and sinusoidal capillarization [23].

Furthermore, possible interactions between 3-HBI and important signaling proteins, including SMAD4, SMAD3, SMAD2, TGF-βR1, and TGF-βR2, have been revealed by molecular docking. According to a comparative binding energy analysis conducted with the SwissDock platform in the Chimera software (version nr: 43.41.1015) framework, 3-HBI might attach to these proteins at locations that overlap with those of recognized ligands, hence preventing their activity. Using the BIOVIA Discovery Studio Visualizer software, the predicted binding sites were further examined, supporting the idea that 3-HBI may interfere with SMAD2, SMAD3, and SMAD4 to impair SMAD signaling (Table 1 and Figure 6). An immunoblot investigation validated these in silico results, confirming that 3-HBI interferes with the canonical TGF-β1-driven signaling cascade by efficiently attenuating the transcriptional expression of fibrosis-associated proteins (Figure 5). This suggests that there is value in the further investigation of 3-HBI for the development of new anti-fibrotic drugs. The binding energy of 3-HBI exhibited good binding scores (−3 to −5 kcal/mol) with target proteins known to be involved in the signaling cascade of SMADs. Molecular docking revealed information about the precise interactions between 3-HBI and proteins in the SMAD2/3 pathway, as there are several proteins that bind in the SMAD signaling pathway This implies that 3-HBI prevents HSC activation by interfering with the TGF-β/SMAD signaling pathway, which might reduce liver fibrosis. While SB431542 (SB), a TGF-β receptor inhibitor, exhibits a TGF-β receptor binding score, SB has a better ΔG value than 3-HBI, of roughly −7.48 kcal/mol. According to earlier reports, β-citronellol showed binding scores of −5.0 to −5.7 kcal/mol with target proteins that are known to be involved in the MAPK signaling cascade, including ephrin type-A receptor 2 and mitogen-activated protein kinase 3 [17]. Furthermore, it has been previously documented that 1-PHE and LDL-receptor-related protein 5, as well as protein kinase cAMP-activated catalytic subunit alpha, have binding scores of ligands and target proteins of liver fibrosis. A favorable interaction was indicated by higher scores of −6.033 and −6.055 Kcal/mol, respectively [16].

There are significant health concerns associated with the hidden development and clinical presentation of liver fibrosis. With the development of targeted therapies and a growing understanding of molecular pathways, there is cautious promise that new therapeutic treatments, such as one based on the anti-fibrotic effect of 3-HBI described here, may soon be developed. The current study was limited to in vitro and in silico methods, which differ from the actual circumstances of liver fibrosis disease in patients. However, in vivo studies, including in animal models, and clinical research are still required to validate these findings in order to fully elucidate the pharmacological effects, toxicity, and mechanisms of action of 3-HBI in the context of liver fibrosis.

## 4. Materials and Methods

### 4.1. Cell Culture

Dulbecco’s modified Eagle’s medium (DMEM; Gibco, Thermo Fisher Scientific, Waltham, MA, USA) was used to cultivate the human HSC cell line LX-2 cells. It was supplemented with 10 millimolar HEPES, 1% penicillin/streptomycin (Gibco–Thermo Fisher Scientific, MA, USA), and 2% fetal bovine serum (Gibco, Carlsbad, CA, USA). The cells were kept at 37 °C with 5% CO_2_ in a humidified incubator. LX-2 cells were provided by Assoc. Prof. Saranyapin Potikanond, M.D., Ph.D., from the Department of Pharmacology, Chiang Mai University, Thailand. In each experiment, 10 ng/mL of TGF-β (Abcam AB50036, Cambridge, UK) was used to generate fibrosis in the cells. Next, as a positive control, the cells were exposed to SB431542(4-(4-(benzo[d][1,3]dioxol-5-yl)-5-(pyridin-2-yl)-1H-imidazol-2-yl)benzamide) (Sigma Aldrich, St. Louis, MO, USA). 3-HBI from Santa Cruz Biotechnology (Dallas, TX, USA) with a purity of ≥90% was used for treatment at concentrations of 5 µg/mL and 10 µg/mL.

### 4.2. Enzyme-Linked Immunosorbent Assay (ELISA)

After cultivating 1 × 10^5^ cells/mL of LX-2 cells, they were treated with 3-HBI for 24 h and stimulated with TGF-β. Following the manufacturer’s instructions, the human IL-6 ELISA pair set kit (cat. SEKB10395), human IL-8 ELISA pair set kit (cat. KA00006), and human MMP-9 ELISA pair set kit (cat. SEKA10327) (Sino Biological Inc., Paoli, PA, USA) were used to measure the levels of IL-6, IL-8, and MMP-9 in the supernatant. Thermo Fisher Scientific’s Varioskan LUX Multimode Microplate Reader was used to detect the absorbance signals.

### 4.3. Real-Time Quantitative Reverse Transcription (qRT-PCR)

The TRIzol reagent (300 μL/well) was added after the culture medium was removed, and the mixture was incubated for 15 min. Following the addition of 50 μL/well of chloroform, the mixture underwent two vortexings before being centrifuged for 15 min at 12,000 rpm. A total of 200 μL of isopropanol was added to the supernatant after it had been moved to a fresh tube. The mixture was then centrifuged for ten minutes at 12,000 rpm. After discarding the supernatant, 200 μL of 75% ethanol was added to the pellet, and centrifugation was performed for 10 min at 12,000 rpm. After discarding the supernatant, the pellet was allowed to air dry and was kept at −20 °C. To synthesize cDNA, 40 μL of RNase-free water was added to each RNA microtube, followed by 2 rounds of vortexing and centrifugation. Next, 4 μL of the Tetro cDNA Synthesis Kit reagent was added to a new PCR reaction tube, and 6 μL of RNA was added. After vortexing and centrifugation, the cDNA synthesis reaction was performed. In real-time qRT-PCR, for the measurement of proinflammatory cytokines, the reagents were prepared, including primers for *ACTA2*, *COL1A1*, *COL4A1*, *TIMP1*, *MMP2*, *MMP9*, *SMAD2*, *SMAD3*, and *SMAD4*. cDNA from the experiments (3 μL) and the SensiFASTTM SYBR^®^ No-ROX Kit reagent (5 μL) were added to a PCR tube, vortexed, and centrifuged for 5 s. Using a CFX96 Touch real-time polymerase chain reaction detection system (Bio-Rad Laboratories, Inc., Hercules, CA, USA), the reaction was measured. One minute of polymerase activation at 95 °C, 45 cycles of denaturation at 95 °C for 15 s, and 1 min of annealing and extension at 60 °C comprised the PCR stage. The human actin beta (ACTB) gene was the housekeeping gene. The 2^−ΔΔCT^ method was used to standardize gene expression in all the data. All the primer sequences are shown in Table 2.

### 4.4. Molecular Docking

A molecular docking experiment was performed to investigate the interactions between 3-HBI and specific proteins involved in HSC activation, a crucial process in liver fibrosis. Three-dimensional protein structures related to HSC activation in hepatic fibrosis were obtained from the RCSB Protein Data Bank (RCSB PDB) [24,25]. Mothers against decapentaplegic homolog 2 (SMAD2) (PDB ID: 6YIA), mothers against decapentaplegic homolog 3 (SMAD3) (PDB ID: 6YIB), mothers against decapentaplegic homolog 4 (SMAD4) (PDB ID: 1DD1), transforming growth factor beta (TGF-β) receptor type 1 (PDB ID: 5E8X), and transforming growth factor beta (TGF-β) receptor type 2 (PDB ID: 5E8V) were the candidate proteins in the TGF-β/SMAD signaling pathway for which crystal structures were accessible in the public domain. The research collaboratory for the structural bioinformatics protein data bank (RCSB PDB) provided the target protein structures. Target proteins of the TGF-β/SMAD signaling pathway were prepared for molecular docking using structure establishment by X-ray crystallography [26,27]. The “.pdb” format was used to obtain all protein files. Using the UCSF Chimera program, all non-protein atoms were removed from the target protein structures to convert them to a monomeric state.

For the docking analysis, the structures that had been processed were saved as “.pdb” files. PubChem provided the chemical structure of 3-HBI (PubChem CID 5363700) [28]. The SwissDock platform (http://www.swissdock.ch, accessed on 1 December 2023) was used to perform protein–ligand blind docking. The in silico docking was carried out using the SwissDock server, which is based on the EADock DSS protein–ligand docking software created by the Swiss Institute of Bioinformatics (SIB) [29], and the visualization and analysis of the docking findings were achieved using UCSF Chimera. The UCSF Chimera alpha software’s DockPrep tool was used to pre-process the protein targets and 3-HBI before docking (version 1.18) [30]. Following docking, the BIOVIA Discovery Studio Visualizer (version 21.1.0.20298, Waltham, MA, USA) was used to visualize the interactions between the ligand and the target proteins in two dimensions.

### 4.5. Western Blot Analysis

Protease and phosphatase inhibitor cocktails (Thermo Fisher Scientific, Waltham, MA, USA) were added to ice-cold RIPA buffer (Bio Basic Inc., Amherst, NY, USA) to lyse the cells for 30 min. The lysates were centrifuged at 12,000 rpm for 30 min at 4 °C, after which the protein concentration of the supernatants was determined using a Bicinchoninic Acid Kit (BCA Kit). Protein samples in equal quantities were separated on a 12% SDS-polyacrylamide gel (PAGE) and electro-transferred to a PVDF membrane (Bio-Rad Laboratories, Inc., Hercules, CA, USA). The membrane was blocked with 5% bovine serum albumin (Cap-ricorn Scientific GmbH, Hesse, Germany) in Tris-buffered saline with Tween 20 (TBST), washed, and incubated with rabbit antibodies against human alpha-smooth muscle actin, p-SMAD2/3, and SMAD2/3 (Cell Signaling Technology, London, UK) for 60 min at room temperature. To detect positive signals, the membrane was thoroughly washed in TSBT and incubated for 60 min at room temperature in horseradish peroxidase (HRP)-linked anti-rabbit IgG secondary antibodies (Cell Signaling Technology, London, UK). Chemiluminescent signals were captured using the ChemiDoc XRS+ Imaging System (Bio-Rad Laboratories, Inc., Hercules, CA, USA); protein bands were visible following incubation for 5 min in the chemiluminescence substrate. The band intensity and protein concentration were established using the Image Studio Lite software v6.0 (LI-COR Corporate, Lincoln, NE, USA).

### 4.6. Statistical Analysis

Three replicates of each in vitro experiment were carried out. The data are presented as the means ± standard deviation (SD). The GraphPad Prism software (version 8.0.1) was used to perform statistical comparisons between the group means using a one-way analysis of variance (ANOVA) and Tukey’s post hoc test for multiple comparisons. A *p*-value of less than or equal to 0.05 was considered to be statistically significant.

## 5. Conclusions

Through crucial processes, 3-HBI inhibits liver fibrosis by disrupting the TGF-β/SMAD signaling pathway. It can downregulate fibrotic markers (e.g., ACTA2, COL1A1, COL4A1, TIMP-1, MMP-2, and MMP-9) and pro-inflammatory cytokines (IL-6 and IL-8). Molecular docking provides information about the interactions between 3-HBI and significant SMAD pathway components with favorable binding energies. These results demonstrate that 3-HBI targets fibrosis-associated signaling pathways, thus providing a promising intervention and treatment for hepatic fibrosis.

## Figures and Tables

**Table 1:** The affinity of 3HBI binding on target proteins in the SMAD pathway.

PDB ID	Protein Name	Binding Free Energy (kcal/mol)	Interacting Amino Acid
1DD1	Mothers against decapentaplegic homolog 4	−5.47	ARG502, GLN410, GLN516, TRP509, VAL426
6YIB	Mothers against decapentaplegic homolog 3	−4.92	ALA352, LEU220, LEU351, TRP405, VAL223, VAL355, VAL409
6YIA	Mothers against decapentaplegic homolog 2	−4.69	ASP304, CYS312, HSD291, PHE290, PHE311
5E8X	Transforming growth factor beta (TGF-β) receptor type 1	−4.57	HSD43, ILE85, LEU87, VAL34, VAL41
5E8V	Transforming growth factor beta (TGF-β) receptor type 2	−3.01	ASN40, GLN41, LYS42, PHE11, PHE126

**Table 2:** Primers for real-time qRT-PCR.

Genes	Description	Forward Primer (3′ → 5′)	Reverse Primer (3′ → 5′)
*ACTA2*	Actin alpha 2, smooth muscle	CATCCTCATCCTCCCTTGAG	ATGAAGGATGGCTGGAACAG
*COL1A1*	Collagen type I alpha 1 chain	CCGGCTCCTGCTCCTCTTAGCG	CGTTCTGTACGCAGGTGATTGGTGG
*COL4A1*	Collagen type IV alpha 1 chain	CCTGGCTTGAAAAACAGCTC	CCCTGCTGAGGTCTGTGAAC
*TIMP1*	TIMP metallopeptidase inhibitor 1	CAAGATGTATAAAGGGTTCCAAGC	TCCATCCTGCAGTTTTCCAG′
*MMP2*	Matrix metallopeptidase 2	AAGTATGGCTTCTGCCCTGA	ATTTGTTGCCCAGGAAAGTG
*MMP9*	Matrix metallopeptidase 9	CGAACTTTGACAGCGACAAG	CACTGAGGAATGATCTAAGCCC
*GAPDH*	Glyceraldehyde-3-phosphate dehydrogenase	ATGACATCAAGAAGGTGGTG	CATACCAGGAAATGAGCTTG′
*SMAD2*	SMAD family member 2	TGCTCTGAAATTTGGGGACTGA	GACGACCATCAAGAGACCTGG
*SMAD3*	SMAD family member 3	ATCGTGAAGCGCCTGCTG	CATCCAGGGACCTGGGGA
*SMAD4*	SMAD family member 4	GCCCGAGCCCAGGTTATC	ACAATGCTCAGACAGGCATCA

## Data Availability

The data presented in this study are available on request from the corresponding author.
